# Sonochemically-Promoted Preparation of Silica-Anchored Cyclodextrin Derivatives for Efficient Copper Catalysis

**DOI:** 10.3390/molecules24132490

**Published:** 2019-07-07

**Authors:** Katia Martina, Federica Calsolaro, Alessio Zuliani, Gloria Berlier, Fernando Chávez-Rivas, Maria Jesus Moran, Rafael Luque, Giancarlo Cravotto

**Affiliations:** 1 Dipartimento di Scienza e Tecnologia del Farmaco, Università di Torino, Via Pietro Giuria 9, 10125 Turin, Italy; 2 Departamento de Quimica Organica, Universidad de Cordoba, Edificio Marie-Curie (C-3), Ctra Nnal IV-A, Km 396 Cordoba, Spain; 3 Dipartimento di Chimica, Università di Torino, Via Pietro Giuria 7, 10125 Turin, Italy; 4 Instituto Politécnico Nacional, ESFM, Departamento de Física, UPALM, Zacatenco, Ciudad de México 07738, Mexico; 5 Peoples Friendship University of Russia (RUDN University), 6 Miklukho-Maklaya str., 117198 Moscow, Russia

**Keywords:** sonochemistry, copper-catalysis, β-cyclodextrin, silica, green chemistry, organic-inorganic hybrid materials, infrared spectroscopy, diffuse reflectance, UV-visible

## Abstract

Silica-supported metallic species have emerged as valuable green-chemistry catalysts because their high efficiency enables a wide range of applications, even at industrial scales. As a consequence, the preparation of these systems needs to be finely controlled in order to achieve the desired activity. The present work presents a detailed investigation of an ultrasound-promoted synthetic protocol for the grafting of β-cyclodextrin (β-CD) onto silica. Truly, ultrasound irradiation has emerged as a fast technique for promoting efficient derivatization of a silica surface with organic moieties at low temperature. Three different β-CD silica-grafted derivatives have been obtained, and the ability of β-CD to direct and bind Cu when CD is bonded to silica has been studied. A detailed characterization has been performed using TGA, phenolphthalein titration, FT-IR, diffuse reflectance (DR), DR UV-Vis, as well as the inductively-coupled plasma (ICP) of the β-CD silica-grafted systems and the relative Cu-supported catalysts. Spectroscopic characterization monitored the different steps of the reaction, highlighting qualitative differences in the properties of amino-derivatized precursors and final products. In order to ensure that the Cu-β-CD silica catalyst is efficient and robust, its applicability in Cu(II)-catalyzed alkyne azide reactions in the absence of a reducing agent has been explored. The presence of β-CD and an amino spacer has been shown to be crucial for the reactivity of Cu(II), when supported.

## 1. Introduction

The design of novel organic-inorganic hybrid systems has achieved considerable success in several fields, including catalysis, photochemistry, biochemistry, and optoelectronics [1]. Numerous innovative studies have enriched the literature on this type of compound, and especially so in recent years [2,3,4,5]. For example, a hybrid inorganic-organic, TiS_2_, and ammonium-cation-based superlattice has recently been described as a promising flexible thermoelectric thin film [6]. Moreover, an innovative system, consisting of a core of BaTiO_3_, covered by PMMA, has been demonstrated to have tailorable dielectric properties [7]. The key feature of this type of system is that its characteristics arise not only from the individual contributions of the phases, but also from the properties of the inner interface, which can also be predominant. The unique active behavior of these hybrid systems is therefore not observed when the single components are used alone [8].

Organic-inorganic hybrid systems can be divided into two classes according to the nature of the organic-inorganic interface [9]. Class I materials possess organic and inorganic components bonded together via weak interactions, such as van der Waals forces, hydrogen bonding, and ionic bonds. Class II compounds are made up of stronger covalent or iono–covalent interactions [9]. Some organic-inorganic hybrid systems of both classes have emerged as a winning combination in the general field of catalysis due to their enhanced heterogeneous properties. In fact, the immobilization and stabilization of organic active sites over inorganic solids has strongly improved the recovery and recyclability characteristics [1,10]. However, class II compounds have attracted much more interest from the field of catalysis due to the higher stability of their hybrid systems. Indeed, class II materials can be used under harsher reaction conditions, whereas class I systems are more sensitive and may be irreversibly damaged. For example, mesoporous silica, such as SBA-15 and MCM-41, has been functionalized with numerous organic ligands for several catalytic applications [10,11,12,13]. The evolution of Class II hybrid components has led to the growth of materials in which catalytically-active coordination-metal complexes are covalently anchored to inorganic substrates [14,15,16,17,18]. Due to the impractical nature of infinitely recycling the metals, research is being directed toward the utilization of more abundant and inexpensive metals, such as iron, cobalt, copper, and nickel, in the place of precious metals [19,20,21,22,23,24]. Specifically, copper catalysis has received a great deal of attention in recent years [25,26,27,28,29]. In fact, copper has undergone thorough investigations in its role as a homogeneous catalyst, and remarkable results have been achieved in many reactions, including oxidations, additions, C-C bond cleavage, and Michael addition reactions [30,31,32,33,34]. Therefore, the development of stable Cu catalysts that can be used in heterogeneous conditions has recently attracted considerable interest. For example, copper nanoparticles that are supported over silica have been studied as efficient catalysts for the hydrogenation of alkynes [35]. Furthermore, Cu that is supported over manganese oxide octahedral molecular sieves has been demonstrated to be an efficient catalyst for the synthesis of imidazoles [36].

**Figure 1:**
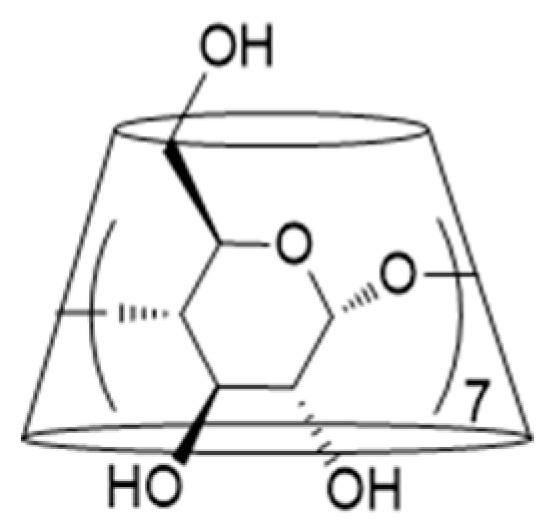
General structure of β-cyclodextrin (β-CD).

The purpose of Cu organic-inorganic hybrid systems resides in the possibility of synthesizing heterogeneous catalysts with the lowest possible metal dimensions in order to increase active surface area. The ambitious major objective in this field is therefore that of producing atomic-scale particles. This can be achieved via the selection of stable ligands for Cu that can be strongly anchored over the various solid supports. β-cyclodextrin (β-CD, Figure 1) has been demonstrated to be an attractive macromolecule for this purpose. β-CD is a cyclic oligosaccharide composed of seven linked D-glucopyranose units and that can be represented as a toroid structure with narrow and wide openings corresponding to the primary and secondary hydroxyl groups, respectively [37]. This peculiar structure means that β-CD can easily encapsulate copper and numerous other compounds [38,39,40]. Several studies have proposed ingenious strategies for supporting Cu/β-CD complexes. For example, Moradi et al. recently stabilized Cu(II)/β-CD complexes over fibrous nanosilica and successfully employed the material as a catalyst in the synthesis of oxazolidinones [41]. Xia and co-workers prepared a magnetically-recoverable catalyst made of Cu(II)/β-CD complexes anchored over ferric oxide and covered by silica [42]. Shafiee et al. reported the preparation of functionalized PEGylated mesoporous silica nanoparticle-graphene oxide as a catalyst for the synthesis of imidazoles [43]. 

Ultrasound (US) has seen widespread use in the promotion of organic syntheses [44,45] and heterogeneous phase reactions [46,47]. In addition, sonochemical techniques have been claimed to have outstanding methodologies for sustainable and green synthesis [48]. Significant effort has been devoted to organic synthesis with supported catalysts and reagents on the surfaces of inexpensive and recyclable mineral supports, such as clay, silica, and alumina [49]. However, only a limited number of publications have made reference to US-promoted silica derivatization. The focus is mainly on Class I derivatives that possess organic and inorganic components. Sonication has been efficiently employed to give silica-based nanocomposites. For example, guanidine acetic acid has been immobilized on the surface of modified Fe_3_O_4_@SiO_2_ [50], and carbon nanotubes have been derivatized with tetraethoxysilane using an ultrasonic sol-gel process [51]. Only a few examples have studied the preparation of Class II derivatives, and an alkoxysilane derivate of thiacalix[4]arene has been efficiently covalently grafted onto silica [52].

Herein, we describe a detailed investigation into the influence of US irradiation on the reaction rate of silica derivatization to obtain CD-grafted silica. A number of routes have been explored and compared with the aim of obtaining this class of peculiar organic-inorganic hybrid materials using a highly-reproducible, efficient synthetic protocol. Commonly-employed silica-grafting procedures entail long reaction times at high temperatures, and the capability of US to shorten reaction times and operate at lower temperatures has been demonstrated. By supporting Cu(II) on the CD-grafted silica, we were able to produce a new copper catalyst for click azido-alkyne cycloadditions. Remarkably, the click 1,3-dipolar cycloaddition was optimized without the addition of a reducing agent because of the presence of CD. Due to the utilization of an abundant metal in Earth’s crust and the exploitation of sonication, the so-produced material could be pointed out as a novel green and sustainable hybrid organic-inorganic catalyst [53].

## 2. Results and Discussion

### 2.1. Synthesis of Copper Supported on Si-NH-CD and Si-DETA-CD

**Scheme 1:**
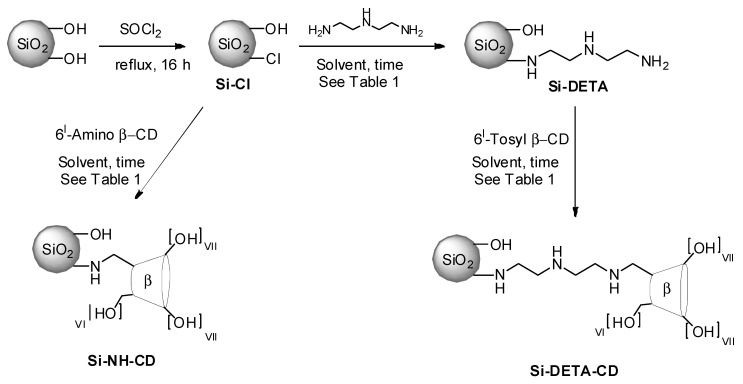
Synthetic schemes for the preparation of Si-NH-CD and Si-DETA-CD.

On the basis of our experience with covalently-functionalized silica [54,55], the modification of silica surfaces via the grafting of organic compounds can lead to improve catalytic activity and may increase sorption capacity. With the aim of obtaining different silica-support-grafted β-CDs for Cu(II) species using a short and reliable synthetic protocol, two different routes were compared. Specifically, β-CD was anchored to silica via a diethylentriamino spacer that can contribute to Cu binding strength, and amino CD was directly grafted onto the silica surface (Scheme 1). Precipitated silica SIPERNAT 320 (Evonik) was used as an inorganic support, with a moderate absorption capacity and BET specific surface area (SSA) of 164 m^2^/g. The sample was characterized by medium particle size, in the range of tenths to hundredths of micrometers, formed by the agglomeration of nanosized (20–50 nm) primary particles. Interparticle porosity resulted in relatively large pore volume (1.2 cm^3^/g). Preliminarily, silica was converted to silica chloride (Si-Cl) in the presence of thionyl chloride, as has already been described in the literature [56,57], and the resulting chloride ions were titrated with a silver nitrate solution in the presence of a potassium chromate solution, used as an indicator. 6^I^ amino-6^I^-deoxy-β-CD was then reacted to obtain the Si-NH-CD derivatives. In the second route, which gives the Si-DETA-CD derivative, 6^I^-tosyl-β-CD was anchored to silica via a diethylentriamino spacer. Chlorinate silica was then reacted with diethylenetriamine (DETA), and a nucleophilic substitution with 6^I^-tosyl-β-CD was performed (Scheme 1).

**Figure 2:**
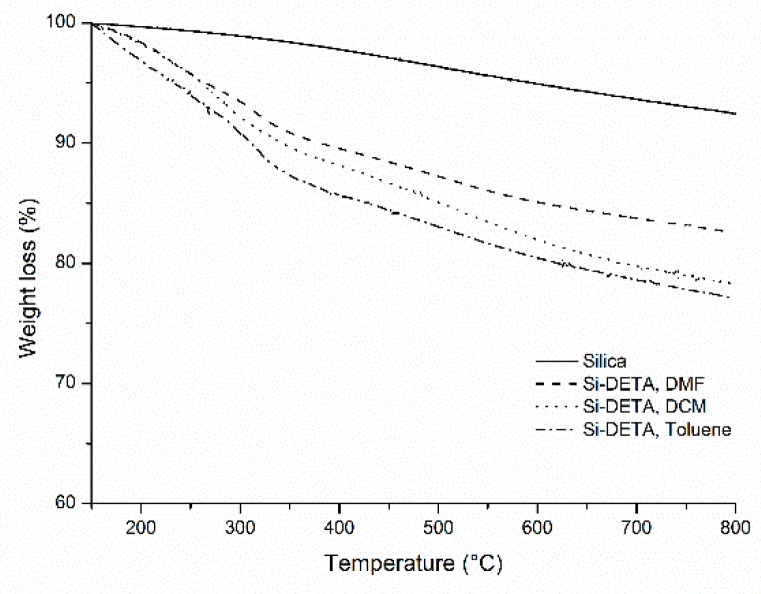
TGA profiles of starting silica and Si-DETA substrates (Table 1, Entries 1–3). The reaction mixture was heated at 60 °C and conventionally stirred for 12 h. Three different solvents were tested. Solid line, starting silica; dashed line, Si-DETA prepared in DMF, Table 1, Entry 2; dotted line, Si-DETA prepared in DCM, Table 1, Entry 1; dashed-dotted line, Si-DETA prepared in toluene, Table 1, Entry 3.

**Figure 3:**
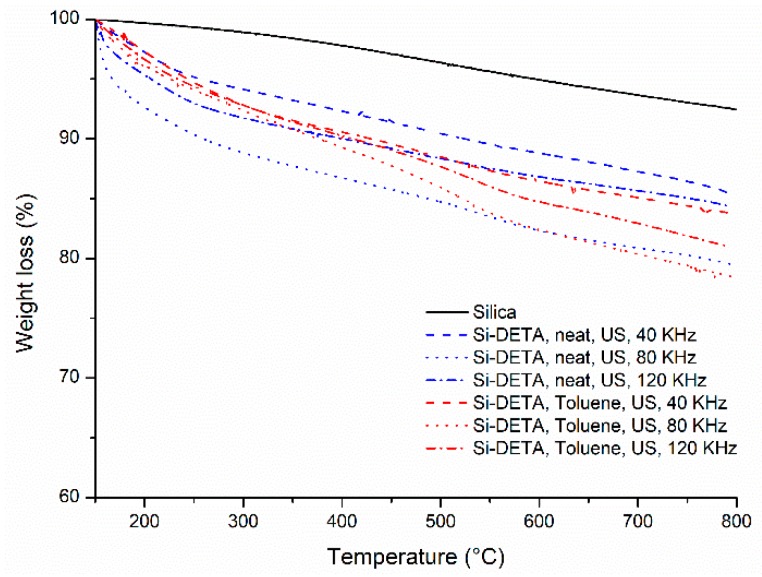
TGA profiles of silica and Si-DETA obtained using US irradiation at different frequencies (40–80–120 kHz), in neat conditions (Table 1, Entries 7–9, blue lines) and in toluene (Table 1, Entries 4–6, red lines). Solid line, starting silica; blue, dashed line, Si-DETA, neat, US 40 kHz, Table 1, Entry 7; blue, dashed-dotted line, Si-DETA, neat conditions, 120 kHz, Table 1, Entry 9; red, dashed line, Si-DETA, toluene, 40 kHz, Table 1, Entry 4; red, dashed-dotted line, Si-DETA, toluene, 120 kHz, Table 1, Entry 6; blue, dotted line, Si-DETA, neat conditions, 80 kHz, Table 1, Entry 8; red, dotted line, Si-DETA, toluene, 80 kHz, Table 1, Entry 5.

In order to optimize the reaction conditions, both schemes were performed under different reaction conditions, as depicted in Table 1. Thermogravimetric analyses (TGA) and IR spectra were carried out to measure the loading and to confirm the identity of the synthesized silica derivatives. TGA allowed the grafting efficacy of every single step to be quantified by assuming that water is the only compound removed from the starting silica by surface dehydroxylation. The curves were all normalized to 150 °C to circumvent any possible solvent influence on yield calculations. When DETA was reacted with silica chloride, a number of solvents were compared under conventional conditions, and a suspension of Si-Cl in toluene, chloroform, and DMF was treated with the triamine derivative at 60 °C for 12 h. As represented in Figure 2, the TGA analyses of Si-DETA showed a higher degree of derivatization when toluene was used as the solvent. The first derivative peak temperature (315 °C) indicates the point of the greatest rate of change on the weight loss curve, and the profile was consistent in all three samples. The synthetic process was also performed under sonication in order to reduce the reaction time and improve functionalization. US irradiation is considered to be a nonconventional means to achieve efficient heating, dispersion, the deagglomeration of solids, and improvements in mass transfer. It offers a facile, versatile synthetic tool for the preparation of nano-/micro-structured materials that are often unavailable via conventional methods. Several different frequencies (40–80–120 kHz) were used and compared when the reaction was repeated under sonication. For the sake of comparison, the reaction was sonicated for 2 and 4 h, and the results obtained in toluene were compared to reactions performed neat in diethylenetriamine as the solvent. As depicted in Table 1, the same degree of substitution that was measured by TGA after 2 h of sonication was achieved after 12 h at 60 °C, while after 4 h of US irradiation, the degree of substitution was maintained without improvement. Figure 3 shows the TGA profiles of Si-DETA batches obtained at a range of different frequencies, either in toluene or neat in 2 h. An increase in weight loss was observed when the reactions were sonicated at 80 kHz. 40 and 120 kHz failed to give the maximum degree of substitution under both sets of reaction conditions.

**Figure 4:**
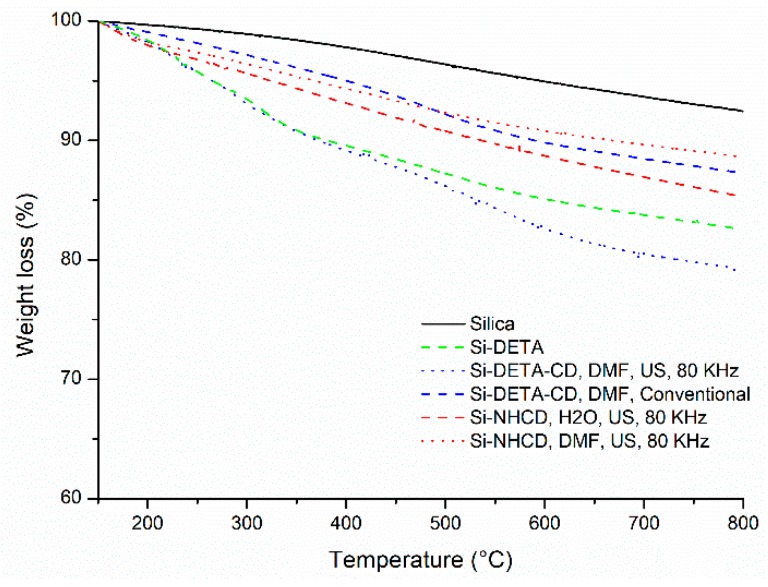
TGA profile of starting silica, Si-DETA (green line), Si-NHCD (red lines), and Si-DETA-CD (blue lines) prepared conventionally or under US irradiation. Solid line, starting silica; red, dotted line, Si-NHCD US irradiation 80 kHz in DMF for 2 h, Table 1, Entry 20; blue, dashed line, Si-DETA-CD conventional heating in DMF at 70 °C for 24 h, Table 1, Entry 16; red, dashed line, Si-NHCD US irradiation 80 kHz in H_2_O for 2 h, Table 1, Entry 21; green, dashed line, Si-DETA; blue, dotted line, Si-DETA-CD US irradiation 80 kHz in DMF for 4 h, Table 1, Entry 17.

When β-CD was grafted onto Si-DETA via the nucleophilic replacement of 6^I^-tosyl β-CD, the reaction was performed conventionally in DMF at 70 °C for 24 h (Entry 16, Table 1). Looking at TGA (Figure 3), we can assume that DETA was acting as the leaving group as the organic moiety on the silica surface was reduced in weight after the reaction if compared to the starting material. The interaction between β-CD and phenolphthalein (Php) was used to achieve the dual aims of measuring the amount of β-CD that maintains its inclusive properties when grafted onto the silica and of investigating the inclusion capacity of the grafted β-CD. In combination with TGA, the amount of β-CD was measured via titration with Php in the buffer solution (pH = 10.5) of 0.008 mM. The amount of grafted β-CD was measured on the basis of UV adsorbance, via interpolation from the standard curve. The change in Php absorbance was recorded on a UV spectrophotometer at 553 nm. When the grafting of CD was performed under conventional heating, by UV adsorbance of the Php solution, we could detect the presence of solid supported β-CD and measure a *w*/*w* % of grafting of 1.3 mg/100 mg Entry 16, Table 1). The same sample analyzed by TGA showed a decrease in total weight loss in the range 150 °C–800 °C if compared to the starting Si-DETA derivative (see Figure 4). Based on this fact, we assumed the instability of the precursor in the reaction condition, as furthermore confirmed by IR analysis described below. Under sonication at 80 kHz, the grafting percentage increased to 3.3% *w*/*w*, and PhP titration confirmed the derivatization data (Entry 17, Table 1). Unfortunately, we observed that the preparation of Si-DETA-CD was not reproducible, and so, we proceeded with the direct grafting of 6^I^ amino β-CD to Si-Cl (Scheme 1).

As described in Table 1, when 6^I^-amino-6^I^-deoxy-β-CD was reacted with Si-Cl for 12 h at 60 °C in DMF, a *w*/*w* % degree of derivatization of 4% was detected by TGA, while PhP titration detected only 0.99% of β-CD, providing an inconclusive answer to the preservations of its properties (Entries 18–19, Table 1). A higher percent of grafting was observed in water. When the reaction was repeated under sonication, the reaction reached the same degree of derivatization in 2 h; after 4 h, any improvement was observed (Entries 20–23, Table 1 Figure 3).

Copper(II) forms a complex with CD in alkaline solution, and the complexes with α- and β-CD have already been isolated and characterized [58]. The efficacy of β-CD to direct and bind Cu when CD is bonded to silica has been already studied, and its catalytic activity as a Lewis acid has been demonstrated [59]. As the literature has focused mainly on the non-covalent bonding of β-CD onto the silica surface [60,61], we have aimed to explore the efficacy of β-CD that is covalently grafted onto silica to chelate Cu(II) and to exploit this system as a catalyst for alkyne-azide cycloaddition (CuAAC) in the absence of a reducing agent.

Si-DETA CD and Si NH CD (Entries 17 and 22, Table 1) were both loaded with Cu(II), and the reaction was performed in a NaOH water solution to obtain a blue-colored catalyst.

### 2.2. Characterization of Copper Supported on Si-NH-CD and Si-DETA-CD

**Figure 5:**
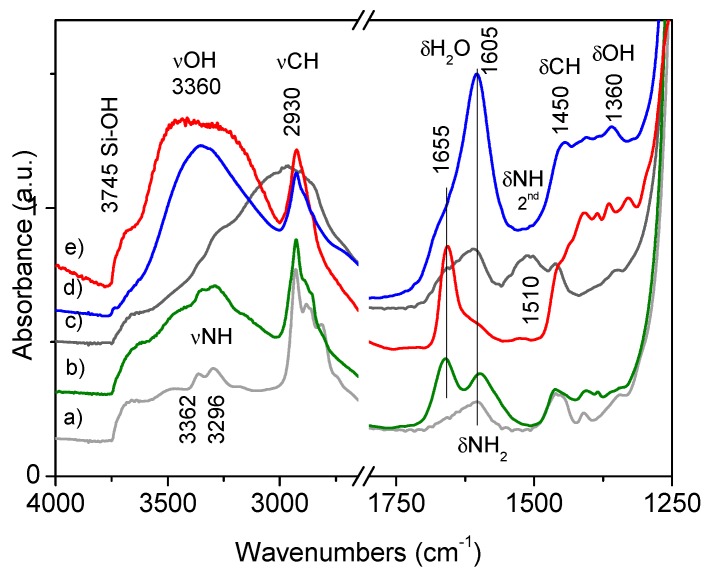
Infrared spectra of: (**a**) Si-diAm; (**b**) Si-diAm-CD-Cu; (**c**) Si-DETA; (**d**) Si-DETA-CD-Cu; (**e**) Si-NH-CD. All samples were outgassed at 80 °C before measurements.

The BET surface area of the functionalized materials was found to be around 110 m^2^/g. The micrometer-sized particles, formed by nanometer-sized primary particles, were preserved after sonication, as observed by scanning electron microscopy (SEM; not reported). Si-DETA, Si-DETA-CD, and Si-NH-CD (Entries 5, 17, and 21, Table 1) were characterized by IR to prove the identity of the grafted silica. The same was done for Si-DETA-CD-Cu and Si-NH-CD-Cu. As shown in Figure 5, the presence of the diethylentriamino spacer on Si-DETA can be observed in Spectrum c. This was characterized by a broad absorption band centered on 3000 cm^−1^, which can be ascribed to hydrogen-bonded Si-OH and N–H groups [62]. The expected υNH bands can be only seen as a modulation of the intense absorption, between 3400 and 3200 cm^−1^. The corresponding weak δNH_2_ mode was vibrating at a similar frequency as the physisorbed water (around 1600 cm^−1^), while the band at 1510 cm^−1^ can be ascribed to the δNH vibrations of the secondary amines. Important changes can be observed in the spectrum (Curve d) after the reaction with 6^I^-tosyl-β-CD (and subsequent Cu inclusion). Namely, the high frequency region was dominated by an intense band centered at 3360 cm^−1^ (υOH), with clear υCH/υCH_2_ bands at 2930 cm^−1^ (shoulder at 2860 cm^−1^). The low frequency region shows the typical bending mode of physisorbed water δH_2_O centered at 1605 cm^−1^ (shoulder at 1662 cm^−1^) and a complex group of bands between 1500 and 1250 cm^−1^ (δCH and δOH). These features can be safely ascribed to the presence of β-CD, even if the assignment of the shoulder at 1662 cm^−1^ to physisorbed water alone is not straightforward. Similar results were obtained with the sample prepared with direct Si-NH-CD (Curve e). The main difference was in the relative intensity of the bands assigned to δH_2_O modes. A comparison of the spectra of β-CD and Cu-CD (not reported) led us to propose that this vibrational mode is sensitive to the presence of the Cu(II) ions included in the cavity and at the rim of the CD cavity. These results thus confirmed the presence of β-CD on the silica in the various preparations. However, the changes in the high frequency region passing from Si-DETA to Si-DETA-CD (Curves c and d) suggested a decrease in the amount of the diethylentriamino spacer, confirming the instability of Si-DETA when submitted to reaction with 6^I^-tosyl-β-CD. The hydroxyl group of CD may act as a nucleophile, and DETA cannot be efficiently and reproducibly washed from the silica surface because of the strong interactions with surface silanol/silanolate groups [63].

**Figure 6:**
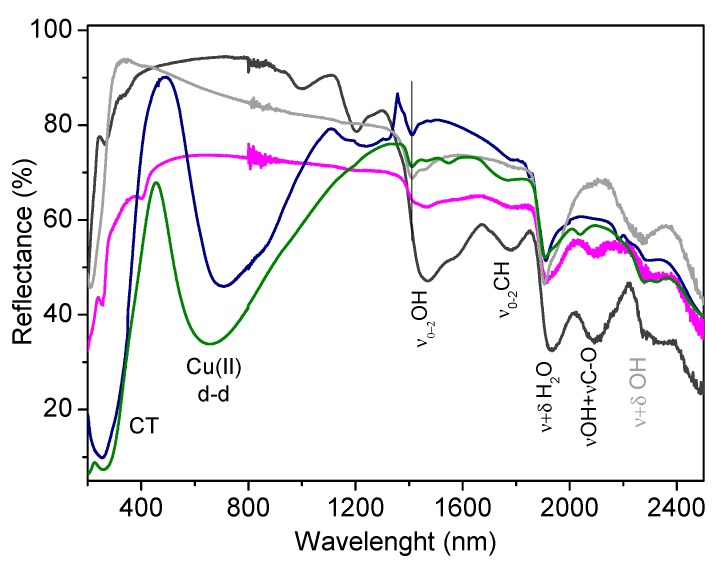
Diffuse reflectance DR UV-Vis-NIR spectra of: SiO_2_ (grey); β-CD (black); Si-NH-CD (pink); Si-NH-Cu-CD (blue); and Si-diAm-Cu-CD (green).

The presence of Cu on the catalysts was monitored by diffuse reflectance UV-Vis-NIR spectroscopy. For example (see Figure 6), we report the spectrum of Si-NH-CD-Cu (blue curve) compared to the spectra of the reference materials (β-CD, SiO_2_, and Si-NH-CD). The spectra can be divided into three regions: (i) UV (200–400 nm), corresponding to O^2−^ → Cu(II) charge transfer (CT) transitions; (ii) visible (400–800 nm), corresponding to the ligand-field d-d transitions of Cu(II) ions; and (iii) NIR (800–2500 nm), where the overtone and combination modes of infrared modes can be observed. The NIR region analysis allows us to further confirm the presence of the CDs that are linked to the silica support and to confirm the involvement of OH groups in the coordination of the Cu ions. Indeed, all the hybrid-material spectra showed signals in this region that were a mixture of the vibrational modes of SiO_2_ (Si-OH overtone υ_0-2 OH_, υ + δ_H2O_/υ + δ_OH_ combination modes of physisorbed water and of Si-OH groups) and those of CD (υ_0-2 OH_, υ + δ_H2O_, υ_0-2 CH_, υ_OH_ + υ_CO_). On the other hand, the UV-Vis region of Si-NH-CD-Cu clearly showed the typical fingerprint of hexacoordinated Cu(II) ions, with a CT component at 260 nm and an intense and broad d-d band at 700 nm [64]. These results were in agreement with the structures proposed in [65], which involved hexacoordinated Cu(II) ions in the cavity and at the rim of the CD cavity, coordinated by H_2_O molecules, OH/O^−^ CD groups, and/or extra ligands.

ICP analyses of Si-NH-CD-Cu and Si-DETA-CD-Cu quantified the Cu content on the silica: at 10.1 mg/g and 7.2 mg/g of Cu, respectively.

### 2.3. Test of the Catalytic Activity of Copper Supported on Si-NH-CD and Si-DETA-CD

**Scheme 2:**
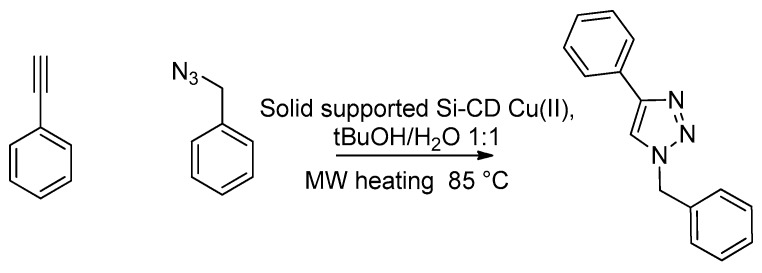
Model reaction for the copper alkyne-azide cycloaddition (CuAAC) test of Si-NH-CD-Cu and Si-DETA-CD-Cu.

Cu(II)-β-CD has been proven to be a reliable, water-soluble, green catalyst for 1,2,3-triazole synthesis without the addition of a reducing agent [40]. Both catalysts were tested in a model CuAAC reaction (Scheme 2).

As depicted in Table 2, the click Cu-catalyzed cyclization of benzyl azide and phenylacetylene was repeated under a number of reaction conditions, and different copper sources were compared. The reaction was performed at 85 °C for 1 h. Silica-supported Si-NHCD-Cu and Si-DETA-CD-Cu showed differing catalytic activities. In fact, only the second acted as an efficient catalyst for CuAAC (Entries 5–6, Table 2). As already observed in the Php titrations, it showed a higher inclusion capability when CD was bound to the silica with a spacer; furthermore, the triamino spacer can stabilize Cu(II) species. Si-DETA-CD-Cu showed high efficiency even when used in very small amounts. However, a reduction of activity was observed when the catalyst amount was decreased to 0.5 mol% (Entries 6–8 Table 2). Cu(II) salts Cu(OAc)_2_ and CuSO_4_ were tested, for the sake of comparison, and the reaction was compared to a click reaction that was catalyzed by a previously-prepared Cu(II)-β-CD complex, and a reaction using a physical mixture of CuSO_4_ and β-CD. Only the Cu(II)-β-CD complex gave satisfactory results, and a 55% yield was recovered. In order to understand the influence of silica and the amino spacer on reaction outcome, Si-Cu and Si-DETA-Cu were tested to confirm that the complex between the copper ions and β-CD played a key role in the performance of the reaction, in which β-CD acted as a ligand for copper and the reducing agent.

### 2.4. Synthesis, Characterization, and Catalytic Activity of Copper Supported on Si-diAm-CD

**Scheme 3:**
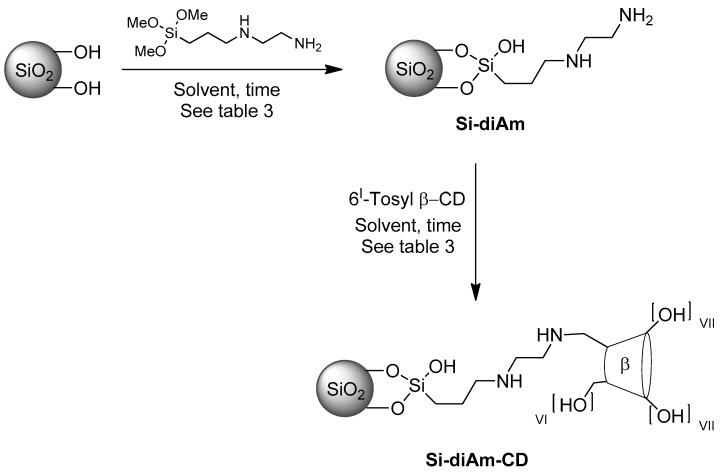
Schematic representation of the Si-diAm-CD synthesis.

In order to obtain a stable silica-supported β-CD derivative with a polyamino spacer, Si-diAm-CD was synthesized on the basis of our previous experience (see Scheme 3).

**Figure 7:**
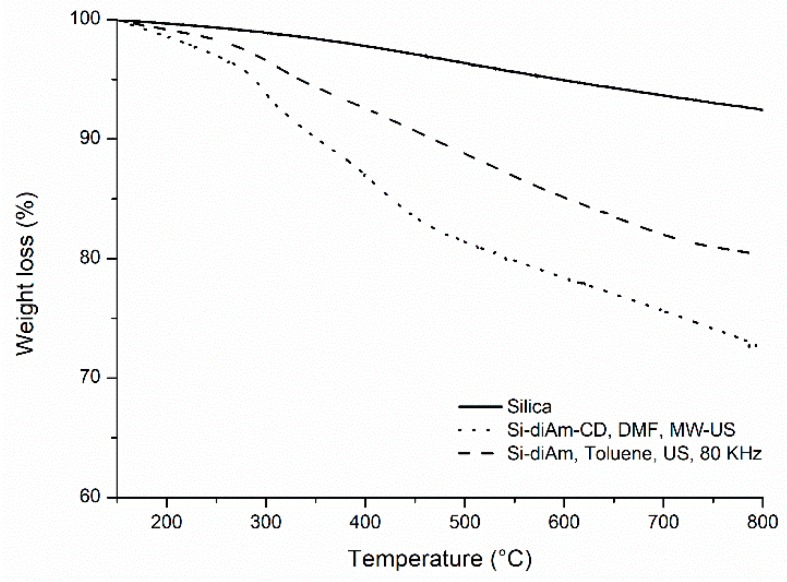
TGA profile of Si-DiAm-CD. (Solid line) Starting silica; (dashed line) Si-DiAm, obtained in US at 80 kHz, 2 h, toluene, Table 3, Entry 4; (dotted line) Si-DiAm-CD, MW-US combined irradiation at 100 °C, 4 h, DMF, Table 3, Entry 9.

Silica was derivatized with 3-(2-aminoethylamino)propyltrimethoxysilane (AEPS) to obtain Si-diAm. As described in Table 3, we can confirm that sonication reduced the reaction time to 2 h, whereas the same degree of substitution was achieved after 36 h at 80 °C under conventional heating and magnetic stirring. No differences in activation was observed between the use of 40 and 80 kHz, and both were very efficient. In order to obtain the efficient grafting of β-CD, 6-*O*-tosyl β-CD was reacted at 60 °C for 60 h, and 4.7% *w*/*w* derivatization was observed by TGA, while 2.8% was measured by Php titration. Sonication at 80 kHz in a US bath reactor was insufficient to obtain a satisfactory grafting percent, because of the low reaction temperature (45–50 °C). An increase in grafting percentage was observed when the reaction was prolonged to six days. The synergic effect of combined MW and sonication was also exploited. Enhancing the silica dispersion, increasing the active surface, and allowing a selective MW irradiation, the combined system permitted obtaining high efficacy of grafting in a short time, 4 h, and a 6.1 *w*/*w*% derivatization was afforded. As depicted in Figure 7, the first derivative TGA profile of Si-diAm indicated that the degradation peak was approximately at 314°; when CD was grafted, two degradation steps were visible, and the peaks were at 298 °C and at 413 °C.

Si-diAm-CD was loaded with Cu (II) in basic conditions, and the catalysts, Si-diAm and Si-diAm-CD, were characterized by IR and DR UV-Vis-NIR.

The IR spectra of Si-diAm and Si-diAm-CD are reported in Figure 2. Si-diAm (Curve a) showed very clear υNH (3362 and 3296 cm^−1^), υCH (2932, 2870, and 2806 cm^−1^), and δNH_2_ bands (ca. 1600 cm^−1^), which were preserved after reaction to graft CD (Si-diAm-CD, Curve b). This proved Si-diAm’s increased stability during the reaction with CD, as compared to Si-DETA derivatization in which the bands were partially displaced (Curves c and d). An analysis of the low-frequency region of the spectra showed the fingerprints of β-CD (δH_2_O, δCH, and δOH). Diffuse reflectance UV-Vis-NIR spectroscopy showed the presence of Cu after the reaction between CuSO_4_ and Si-diAm-CD, and the typical fingerprint of hexacoordinated Cu(II) ions (d-d band at 650 nm and CT at 260 nm) can be observed (Figure 5). ICP analysis showed a loading of 15.5 mg/g of copper.

When catalytic activity was tested in a model reaction (CuAAC of benzyl azide and phenyl acetylene), it was possible to evaluate the high catalytic activity of Si-DETA-CD-Cu. A slightly higher amount of catalyst was employed in order to obtain full conversion, and the reaction was successful in the presence of 4 mol% of catalyst. Alkylic and arylic azido derivatives, as well as alkynes, were tested to prove not only the efficacy, but also the versatility of the system (See Table 4). Full conversion was observed, and the product was isolated without purification.

## 3. Materials and Methods

### 3.1. Materials

All commercially-available reagents and solvents were purchased from Sigma-Aldrich (Milan, Italy) and used without further purification. SIPERNAT 320 amorphous silica was supplied by Evonik Degussa (Essen, Germany). The synthesis of 6^I^-amino-6^I^-deoxy-β-CD and 6^I^-*O*-*p*-Toluenesulfonyl-β-CD was performed following the published synthetic procedure [66]. β-CD was provided by Wacker Chemie (München, Germany). US irradiation at 40–80–120 kHz was performed in highly efficient bath reactors supplied by Weber Ultrasonics GmbH (Karlsbad-Ittersbach, Germany). The transducers’ efficiency was monitored by measurements with a cavitometer. Owing to the size reduction of cavitation bubbles moving from 40, to 80, to 120 kHz, a progressive decrease of the measured cavitation intensity was observed.

**Figure 8:**
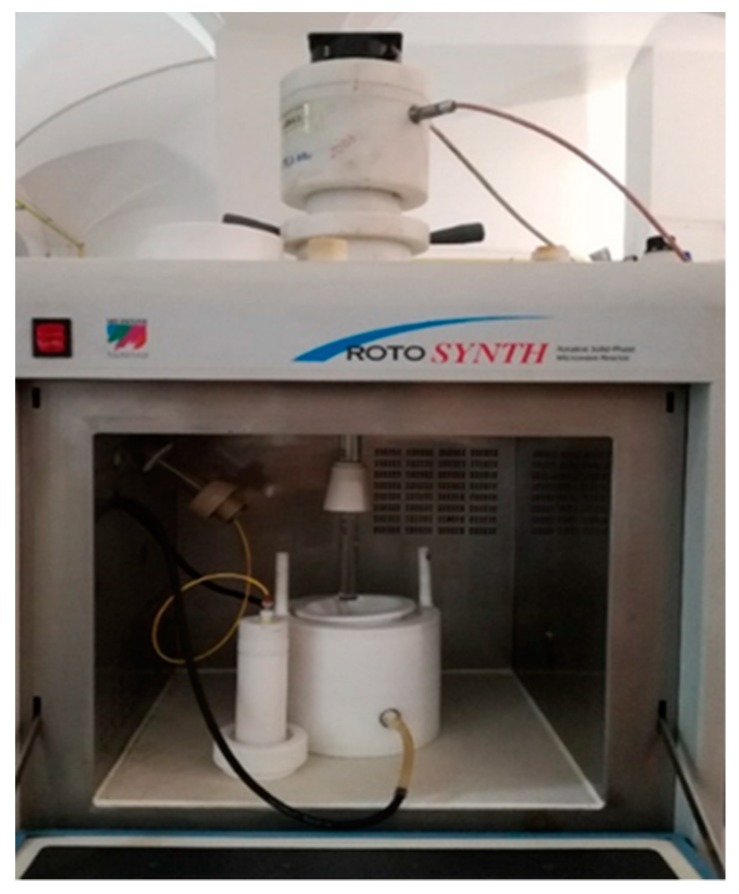
Combined MW/US device.

The reactions were carried out in a combined microwave (MW)/US system [45] designed in our laboratory using a probe equipped with a horn made in Pyrex inserted in an MW cavity (RotoSynth, Milestone, Bergamo) (Figure 8).

Thermogravimetric analyses were performed using a thermogravimetric analyzer TGA 4000 (PerkinElmer, MA, USA) at 10 °C min^−1^ operating with alumina crucibles that contained 10−20 mg of sample. The analyses were performed under an argon atmosphere at a starting temperature of 50 °C and an end temperature of 800 °C. Total mass loss was attributed to the functional groups that were covalently attached to the sidewalls. UV-Vis absorption spectra were measured on a dual-beam spectrophotometer (Agilent Technologies Cary 60, G6860AA, Santa Clara, CA, USA) equipped with a 1-cm path length quartz cuvette. Elemental analyses were performed on an EA 1108 (Fison Instruments). Reactions were carried out in a professional MW reactor (Monowave 400/200, Anton Paar GmbH, Graz, AT) using reaction vial G10. Reactions were monitored by TLC on Merck 60 F254 (0.25 mm) plates (Milan, Italy), which were visualized by UV inspection. GC-MS analyses were performed in a GC Agilent 6890 (Agilent Technologies, Santa Clara, CA, USA) that was fitted with a mass detector Agilent Network 5973, using a 30-m capillary column, i.d. of 0.25 mm, and a film thickness of 0.25 μm. GC conditions were: injection split 1:10, injector temperature 250 °C, detector temperature 280 °C. The gas carrier was helium (1.2 mL/min), and temperature program was from 50 °C (5 min) to 100 °C (1 min) at 10 °C/min, to 230 °C (1 min) at 20 °C/min, and to 300 °C (5 min) at 20 °C/min. HRMS was determined using a MALDI-TOF mass spectra (Bruker Ultraflex TOF mass spectrometer, Milan, Italy). The cations were determined with a Perkin Elmer Optima 7000 (Perkin Elmer, Norwalk, Connecticut, USA) inductively-coupled plasma-optical emission spectrometer (ICP-OES).

### 3.2. Catalyst Preparation

#### 3.2.1. Preparation of Chlorinate Silica 

Ten-point-five milliliter of SOCl_2_ were added dropwise to 1 g of silica SIPERNAT 320. The mixture was left stirring under reflux, 16 h. The suspension was filtered, and the powder was washed with chloroform and dried under a vacuum [56].

##### Si-Cl Titration

The amount of chloride present in the sample was determined via argentometric titration. The Mohr method was followed. The sample solution was titrated against a solution of silver nitrate of known concentration. Chloride ions reacted with silver(I) ions to give insoluble silver chloride (1):

Ag^+^ (aq) + Cl^−^ → AgCl (s) (1)

One gram of Si-Cl was dispersed in 100 mL of a NaHCO_3_ solution (0.005 M). The solution was stirred for 1 h at room temperature. Potassium chromate was used as an indicator, giving red silver chromate after all the chloride ions had reacted:

2 Ag^+^ (aq) + CrO_4_^2−^ (aq) → Ag_2_CrO_4_ (s) (2)

Three hundred ninety eight microliters of K_2_CrO_4_ 0.25 M were added, and the solution was titrated with AgNO_3_ 0.1 M (2) to obtain the amount of chlorosilyl groups on the silica surface (~0.9 mmol g^−1^).

#### 3.2.2. Preparation of Si-DETA

Diethylenetriamine (0.500 mL) was dissolved in 0.500 mL of solvent (see Table 1, Entries 1–3), and Si-Cl was added (0.100 g). The suspension was heated under stirring in an oil bath (60 °C for 12 h). When the reaction was performed in a US bath reactor (40, 80, or 120 kHz, power 200 W), the suspension was sonicated for 2–4 h in toluene or in diethylenetriamine (see Table 1, Entries 4–15). The modified silica was then filtered, washed with water, methanol, and chloroform, and dried under a vacuum at room temperature for 12 h.

#### 3.2.3. Preparation of Si-DETA-CD

6-monotosyl-CD (0.100 g, 0.077 mmol) was dissolved in DMF (1.7 mL), and Si-DETA (0.100 g) was added (Table 1, Entry 16). The suspension was heated to 70 °C under magnetic stirring for 24 h. When the reaction was performed under sonication, the suspension reacted in 4 h (power 200 W, frequency 80 kHz) (Table 1, Entry 17). Silica was filtered, washed with water, methanol, and chloroform, and dried under vacuum at room temperature for 12 h.

#### 3.2.4. Preparation of Si-NH-CD

6^I^-amino-6^I^-deoxy-β-CD (0.163 g, 0.14 mmol) was dissolved in DMF (2 mL) or water, and then, chlorinate silica (0.100 g) and pyridine (0.332 mL) were added (Table 1, Entries 18–23). The suspension was either conventionally stirred at 60 °C for 12 h or was sonicated in a US bath reactor at 80 kHz (power 200 W) for 2–4 h. Modified silica was filtered, washed with water, methanol, and chloroform, and dried under a vacuum at room temperature for 12 h.

#### 3.2.5. Preparation of Si-DiAm

3-(2-Aminoethylamino)propyltrimethoxysilane (0.040 mL) was dissolved in toluene (1 mL), and silica SIPERNAT 320 (0.100 g) was added. The suspension was either heated under stirring in an oil bath (80 °C for 36 h) or the reaction was performed in a US bath reactor (power 200 W, comparing 40 and 80 kHz as frequencies), as depicted in Table 2 (Entries 1–5). Silica was filtered, washed with toluene and chloroform, and dried under a vacuum at room temperature for 12 h.

#### 3.2.6. Preparation of Si-DiAm-CD

6^I^-*O*-*p*-Toluenesulfonyl-β-CD (0.100 g, 0.077 mmol) was dissolved in DMF (1.5 mL), and Si-DiAm (0.100 g) was added. The suspension was either heated to 60 °C and stirred for 60 h or sonicated (4 h, power 200 W, frequency 80 kHz) (Entries 6–9 Table 3). The same procedure was repeated under MW and US combined irradiation: 1 g of Ts-CD was dissolved in 15 mL of DMF, and 1 g of Si-DiAm was added. The suspension was heated at 100 °C for 4 h (average MW power 20 W, average US power 35 W). After cooling to room temperature, the modified silica was filtered, washed with water, methanol, and chloroform, and dried under a vacuum at room temperature for 12 h.

#### 3.2.7. Cyclodextrin-Supported Silica and Copper Complexation

For CD-supported silica (0.100 g), either Si-NH-CD, Si-DETA-CD, or Si-diAm-CD were dispersed in 0.675 mL of NaOH 0.5 M. The suspension was stirred for 1 min by sonication, and 0.719 mL of CuSO_4_ 0.08 M were added dropwise. The mixture was sonicated for 10 s in an US bath reactor and was then filtered and washed thoroughly with water and methanol and, finally, dried under a vacuum at room temperature for 12 h.

### 3.3. Click Chemistry Reaction

Azide (0.0676 mmol, 1 eq) and terminal alkyne (1 eq) were dissolved in 0.500 µL of H_2_O: tBuOH (1:1). Solid-supported catalyst Si-NHCD-Cu, Si-DETA-CD-Cu, or Si-diAm-CD-Cu was added as described in Table 4. The reaction was heated up at 85 °C, for 1 h or under MW irradiation (85 °C) for 20 min. The resulting mixture was filtered and washed with methanol and chloroform. The solvent was removed under vacuum to afford the triazole. All products were confirmed by ^1^H NMR and GC-MS.

### 3.4. Physico-Chemical Characterization

The specific surface area (SSA) of the samples was measured by N_2_ adsorption isotherms at liquid nitrogen temperature using an ASAP 2020 physisorption analyzer (Micromeritics). The SSA was calculated by the Brunauer–Emmett–Teller (BET) method in the p/p^0^ 0.05–0.3 interval. Before measurements, the samples were outgassed at room temperature overnight.

Infrared spectra were recorded on a BRUKER FTIR-66 spectrophotometer with a resolution of 2 cm^−1^, using a DTGS detector. Measurements were carried out using a home-made cell allowing in situ thermal treatment and room temperature measurement. Thin self-supporting pellets for transmission measurements (around 10 mg/cm^2^) were prepared with a hydraulic press. Before the measurements, the samples were outgassed at 80 °C for 2 h in the same cell used for the measurements. β-CD and Cu-CD were measured after dilution in KBr, without thermal treatment (spectra not reported).

UV-Vis-NIR spectra were recorded in the 2500–200-nm range at 1-nm resolution on a Cary 5000 UV-Vis-NIR spectrophotometer (Agilent) equipped with a diffuse reflectance attachment with an integrating sphere coated by BaSO_4_. Prior to each measurement, a baseline spectrum was collected by using Teflon as a reference. Spectra are reported as relative reflectance (R%), defined as:

R_%_ = R_sample_/R_reference_ × 100 (3)

## 4. Conclusions

In conclusion, the ability of sonication to speed up the synthetic procedures for silica grafting was demonstrated. New β-CD grafted silicas were synthesized and fully characterized. The influence of US at different frequencies (40–80 and 120 KHz) was studied and compared to conventional conditions. MW combined with US was also employed in grafting β-CD to previously derivatized silica. The influence that β-CD had on directing and activating Cu(II) on the silica surface for CuAAC reactions, in absence of a reducing agent, was studied and confirmed. Low cost, green, fast, and efficient procedures can be exploited to obtain Cu-β-CD-grafted silica with polyamino spacers under sonication. The catalyst showed excellent performance in CuAAC reactions without the addition of a reduction agent.

## Figures and Tables

**Table 1:** Synthesis of grafted silica.

Entry	Product	Reaction Condition	Time	Loading (*w*/*w* %)	Loading (μmol/g)
1	Si-DETA	Si-Cl, DETA, DCM, reflux	12 h	14 ^(a)^	1.37 × 10^3 (c)^
2	Si-DETA	Si-Cl, DETA, DMF, 60 °C	12 h	10 ^(a)^	0.96 × 10^3 (c)^
3	Si-DETA	Si-Cl, DETA, toluene, 60 °C	12 h	15 ^(a)^	1.49 × 10^3 (c)^
4	Si-DETA	Si-Cl, DETA, toluene, US, 40 kHz	2 h	9 ^(a)^	0.87 × 10^3 (c)^
5	Si-DETA	Si-Cl, DETA, toluene, US, 80 kHz	2 h	14 ^(a)^	1.35 × 10^3 (c)^
6	Si-DETA	Si-Cl, DETA, toluene, US, 120 kHz	2 h	11.5 ^(a)^	1.12 × 10^3 (c)^
7	Si-DETA	Si-Cl, DETA, neat, US, 40 kHz	2 h	7.1 ^(a)^	0.69 × 10^3 (c)^
8	Si-DETA	Si-Cl, DETA, neat, US, 80 kHz	2 h	13 ^(a)^	1.26 × 10^3 (c)^
9	Si-DETA	Si-Cl, DETA, neat, US, 120 kHz	2 h	8.2 ^(a)^	0.79 × 10^3 (c)^
10	Si-DETA	Si-Cl, DETA, toluene, US, 40 kHz	4 h	10 ^(a)^	0.97 × 10^3 (c)^
11	Si-DETA	Si-Cl, DETA, toluene, US, 80 kHz	4 h	14.3 ^(a)^	1.12 × 10^3 (c)^
12	Si-DETA	Si-Cl, DETA, toluene, US, 120 kHz	4 h	10.9 ^(a)^	0.97 × 10^3 (c)^
13	Si-DETA	Si-Cl, DETA, neat, US, 40 kHz	4 h	8 ^(a)^	0.77 × 10^3 (c)^
14	Si-DETA	Si-Cl, DETA, neat, US, 80 kHz	4 h	13.5 ^(a)^	1.26 × 10^3 (c)^
15	Si-DETA	Si-Cl, DETA, neat, US, 120 kHz	4 h	9 ^(a)^	0.88 × 10^3 (c)^
16	Si-DETA-CD	Si-DETA, 6-tosyl β-CD, DMF, 70 °C	24 h	1.3 ^(b)^	11 ^(d)^
17	Si-DETA-CD	Si-DETA, 6-tosyl β-CD, DMF, US, 80 kHz	4 h	3.3 ^(a)^–3.4 ^(b)^	30 ^(c,d)^
18	Si-NH-CD	Si-Cl, 6-amino β-CD, DMF, 60 °C	12 h	4.02 ^(a)^–0.99 ^(b)^	35 ^(c)^
19	Si-NH-CD	Si-Cl, 6-amino β-CD, H_2_O, 60 °C	12 h	2.6 ^(a)^–0.81 ^(b)^	20 ^(c)^
20	Si-NH-CD	Si-Cl, 6-amino β-CD, DMF, US, 80 kHz,	2 h	3.81 ^(a)^–0.68 ^(b)^	30 ^(c)^
21	Si-NH-CD	Si-Cl, 6-amino β-CD, H_2_O, US, 80 kHz,	2 h	7.12 ^(a)^–0.60 ^(b)^	60 ^(c)^
22	Si-NH-CD	Si-Cl, 6-amino β-CD, DMF, US, 80 kHz,	4 h	3.5 ^(a)^–0.67 ^(b)^	30 ^(c)^
23	Si-NH-CD	Si-Cl, 6-amino β-CD, H_2_O, US, 80 kHz,	4 h	6.8 ^(a)^–0.68 ^(b)^	60 ^(d)^

^1^ Preparation of Si-DETA: Si-Cl (0.00 g), solvent (0.500 mL), and diethylenetriamine (0.500 mL). Preparation of Si-DETA-CD: Si-DETA (0.100 g), DMF (1.7 mL), and 6^I^-tosyl-β-CD (0.100 g). Preparation of Si-NHCD: Si-Cl (0.100 g), solvent (2 mL), and 6-amino β-CD (0.163 g). (a) The *w*/*w* % grafting was measured on the basis of TGA; (b) the *w*/*w* % grafting was based on phenolphthalein (Php) titration; (c) mmol/g values were measured on the basis of TGA considering the organic moiety anchored to the silica surface; (d) mmol/g of β-CD based on PhP titration.

**Table 2:** Panel test of the CuAAC reaction.

Entry	Catalyst, Cu mol%	Yield (%) ^(a)^
1	Cu(OAc)_2_, 5 mol%	14
2	CuSO_4_, 5 mol%	0
3	β-CD-CuSO_4_, 5 mol% (physical mixture)	0
4	β-CD-Cu, 5 mol% (complex)	55
5	Si-NHCD-Cu, 4 mol%, (17 mg)	5
6	Si-DETA-CD-Cu, 4 mol% (24 mg)	>99
7	Si-DETA-CD-Cu, 2 mol% (12 mg)	>99
8	Si-DETA-CD-Cu, 1 mol% (6 mg)	>99
9	Si-DETA-CD-Cu, 0.5 mol% (3 mg)	80
10	Si-DiAm-CD, 4 mol% (11 mg)	>99
11	Si-DiAm-CD, 2 mol% (5.5 mg)	65
12	Silica-Cu (12 mg)	4
13	Si-DETA-Cu (12 mg) ^a^	0

Reaction conditions: benzyl azide (0.0676 mmol, 1 eq), phenylacetylene (1 eq), H_2_O:tBuOH (1:1; 500 µL), 85 °C, 1 h, or MW 85 °C 20 min. (a) Determined by GC-MS.

**Table 3:** Synthesis of Si-diAm-CD.

Entry	Product	Reaction Condition	Time	Loading (*w*/*w* %)	Loading (μmol/g)
1	Si-diAm	Silica, AEPS, toluene, 80 °C	6 h	6.5 ^(a)^	290 ^(c)^
2	Si-diAm	Silica, AEPS, toluene, 80 °C	36 h	13.6 ^(a)^	610 ^(c)^
3	Si-diAm	Silica, AEPS, toluene, US, 40 kHz	2 h	14 ^(a)^	640 ^(c)^
4	Si-diAm	Silica, AEPS, toluene, US, 80 kHz	2 h	12 ^(a)^	540 ^(c)^
5	Si-diAm	Silica, AEPS, toluene, US, 80 kHz	4 h	13 ^(a)^	600 ^(c)^
6	Si-diAm-CD	Si-diAm, 6^I^-tosyl-β-CD, DMF, 60 °C	60 h	4.7 ^(a)^–2.15 ^(b)^	42 ^(c)^
7	Si-diAm-CD	Si-diAm, 6^I^-tosyl-β-CD, DMF, 60 °C	6 days	6.1 ^(a)^–2.8 ^(b)^	54 ^(c)^
8	Si-diAm-CD	Si-diAm, 6^I^-tosyl-β-CD, DMF, US, 80 kHz	4 h	1.7 ^(a)^–1.18 ^(b)^	15 ^(c)^
9	Si-diAm-CD	Si-diAm, 6^I^-tosyl-β-CD, DMF, MW/US, 100 °C	4 h	6.1 ^(a)^–2.09 ^(b)^	54 ^(c)^

(a) Measured by TGA; the weight loss was calculated on the basis of the starting material (see Figure 6); (b) the *w*/*w* % grafting was based on PhP titration; (c) mmol/g of organic moiety grafted on the silica surface.

**Table 4:** Synthetic results of conventional Cu-supported catalyst.

Entry	Alkyne	Azide	Product	Yield (%)
	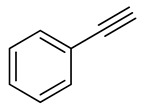	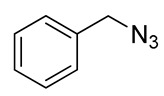	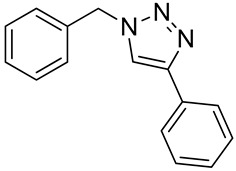	99 ^(a)^
	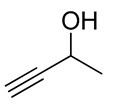	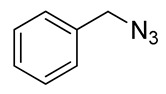	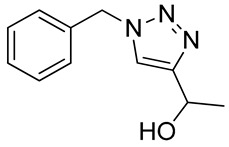	95 ^(a)^
	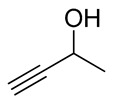	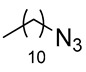	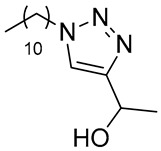	92 ^(a)^
	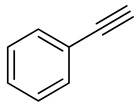	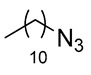	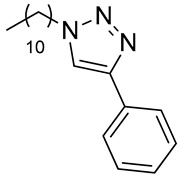	89 ^(a)^

Reaction conditions: azide (0.0676 mmol, 1 eq), terminal alkyne (1 eq), H_2_O (250 µL), tBuOH (250 µL), 11 mg of catalyst (4 mol %), MW heating at 85 °C, 1 h. (a) Yields determined by GC-MS.
